# Supplementary material for: A Likelihood Approach for Real-Time Calibration of Stochastic Compartmental Epidemic Models
Source: PLoS Comput Biol. 2017 Jan 17;13(1):e1005257. doi: 10.1371/journal.pcbi.1005257 (PMC5240920; doi:10.1371/journal.pcbi.1005257)
Supplement: S1 File — (TAR.GZ) [file pcbi.1005257.s014.tar.gz › HSPH_Online-SI-Revision/IPoi09/n10-extreme/n10-extreme_BENCH2_summary.pdf]

peak −8	3w spec	3w cum	5w spec	5w cum	end	3w spec LOG	3w cum LOG	5w spec LOG	5w cum LOG	end LOG
mean	147.4	188.2	75.1	104.5	24.1	0.8	0.8	0.9	0.8	0.6
median	88.9	91.2	76.	87.4	16.1	0.6	0.6	0.5	0.6	0.2
quantile .1	52.9	55.6	40.3	49.8	6.8	0.4	0.4	0.2	0.3	0.
quantile .9	272.	308.1	99.8	201.5	50.2	1.7	1.4	2.1	0.3	1.4
37	38	31	33	28						

peak −4	3w spec	3w cum	5w spec	5w cum	end	3w spec LOG	3w cum LOG	5w spec LOG	5w cum LOG	end LOG
mean	26.1	36.	24.9	20.5	13.1	0.2	0.2	0.2	0.1	0.1
median	24.	34.5	19.9	18.6	10.7	0.1	0.2	0.1	0.1	0.
quantile .1	14.7	22.9	13.1	11.4	3.7	0.1	0.1	0.1	0.1	0.
quantile .9	39.3	54.9	40.1	31.1	24.6	0.2	0.2	0.2	0.1	0.1
42	38	37	40	45						

peak	3w spec	3w cum	5w spec	5w cum	end	3w spec LOG	3w cum LOG	5w spec LOG	5w cum LOG	end LOG
mean	33.	24.7	48.5	27.9	34.1	0.2	0.1	0.2	0.1	0.2
median	30.	22.3	42.9	25.2	31.5	0.1	0.1	0.2	0.1	0.1
quantile .1	22.8	15.3	36.2	16.1	24.8	0.1	0.1	0.1	0.1	0.1
quantile .9	45.2	37.4	64.8	41.2	46.5	0.3	0.2	0.4	0.1	0.3
46	47	42	48	45						

peak +4	3w spec	3w cum	5w spec	5w cum	end	3w spec LOG	3w cum LOG	5w spec LOG	5w cum LOG	end LOG
mean	63.	54.2	89.3	58.7	65.5	0.2	0.2	0.3	0.2	0.2
median	54.5	48.2	65.	51.	53.2	0.2	0.2	0.2	0.2	0.2
quantile .1	42.3	38.	46.6	39.4	43.1	0.2	0.1	0.2	0.1	0.2
quantile .9	97.	79.8	147.5	89.4	98.9	0.3	0.3	0.5	0.1	0.3
40	42	39	43	39						

peak +8	3w spec	3w cum	5w spec	5w cum	end	3w spec LOG	3w cum LOG	5w spec LOG	5w cum LOG	end LOG
mean	103.4	94.4	159.	98.3	108.3	0.3	0.3	0.3	0.3	0.3
median	68.8	67.2	92.9	69.6	72.7	0.2	0.2	0.3	0.2	0.2
quantile .1	51.1	48.6	53.7	49.3	50.2	0.2	0.2	0.2	0.2	0.2
quantile .9	190.7	181.8	207.9	176.2	210.2	0.4	0.4	0.7	0.2	0.4
36	38	40	38	36	{}	{}	{}	{}	{}	{}
